# Feeding methods for children with cleft lip and/or palate: a systematic review^☆^

**DOI:** 10.1016/j.bjorl.2015.10.020

**Published:** 2016-03-02

**Authors:** Giesse Albeche Duarte, Ramon Bossardi Ramos, Maria Cristina de Almeida Freitas Cardoso

**Affiliations:** a Universidade Federal de Ciências da Saúde de Porto Alegre, Programa de Pós-graduação em Ciências da Reabilitação – Linha Musculoesquelética, Porto Alegre, RS, Brazil; b Hospital de Clínicas de Porto Alegre, Porto Alegre, RS, Brazil

**Keywords:** Cleft lip, Cleft palate, Feeding methods, Breastfeeding, Swallowing disorders, Fenda labial, Fissura palatina, Métodos de alimentação, Aleitamento materno, Transtornos de deglutição

## Abstract

**Introduction:**

Feeding difficulties in children with cleft lip and palate (CLP) are frequent and appear at birth due to impairment of sucking and swallowing functions. The use of appropriate feeding methods for the different types of cleft and the period of the child's life is of utmost importance for their full development.

**Objective:**

Review studies comparing feeding methods for children with CLP, pre- and postoperatively.

**Methods:**

The search covered the period between January 1990 and August 2015 in the PubMed, LILACS, SciELO, and Google Scholar databases using the terms: cleft lip or cleft palate and feeding methods or breastfeeding or swallowing disorders and their synonyms. This systematic review was recorded in PROSPERO under number CRD42014015011. Publications that compared feeding methods and published in Portuguese, English, and Spanish were included in the review. Studies with associated syndromes, orthopedic methods, or comparing surgical techniques were not included.

**Results:**

The three reviewed studies on the period prior to surgical repair showed better feeding performance with three different methods: squeezable bottle, syringe, and paladai bottle. Only one study addressed the postoperative period of cleft lip and/or palate repair, with positive results for the feeding method with suction. Likewise, the post-lip repair studies showed better results with suction methods. After palatoplasty, two studies showed better performance with alternative feeding routes, one study with suction method, and one study that compared methods with no suction showed better results with spoon.

**Conclusion:**

The studies show that prior to surgical repair, the use of alternative methods can be beneficial. In the postoperative period following lip repair, methods with suction are more beneficial. However, in the postoperative period of palatoplasty, there are divergences of opinion regarding the most appropriate feeding methods.

## Resumo

**Introdução:**

As dificuldades de alimentação em crianças com fissura labiopalatina (FLP) são frequentes e surgem logo ao nascimento, devido ao comprometimento das funções de sucção e deglutição. A utilização de métodos de alimentação adequados aos diferentes tipos de fissura e ao momento da vida da criança é primordial para seu pleno desenvolvimento.

**Objetivo:**

Revisar estudos que compararam métodos de alimentação para crianças com FLP antes da correção cirúrgica e no pós-operatório.

**Método:**

A busca compreendeu o período entre janeiro de 1990 e agosto de 2015, nas bases de dados PubMed, LILACS, Scielo e Google Acadêmicos e utilizando os termos: Fenda Labial ou Fissura Palatina e Métodos de Alimentação ou Aleitamento Materno ou Transtornos de deglutição e seus sinônimos. Esta revisão sistemática foi registrada no PROSPERO sob o número CRD42014015011. Foram incluídas publicações comparando métodos de alimentação nos idiomas português, inglês e espanhol. Pesquisas com síndromes associadas, métodos ortopédicos ou comparando técnicas cirúrgicas não foram incluídas.

**Resultados:**

Os três estudos revisados sobre o período que antecede a correção cirúrgica apresentaram melhor desempenho na alimentação com três diferentes métodos: mamadeira compressível, seringa e paladai. Um único estudo abordou o pós-operatório de fissura de lábio e/ou palato, apresentando resultados positivos para a alimentação com método com sucção. Da mesma forma, no pós-queiloplastia os estudos mostraram melhores resultados com métodos com sucção. Após a palatoplastia, dois estudos apresentaram melhor desempenho com via alternativa de alimentação; um estudo com método com sucção; e outro que comparou métodos sem sucção apresentou melhores resultados com colher.

**Conclusão:**

Os estudos mostram que, antes da correção cirúrgica, a utilização de métodos alternativos pode apresentar benefícios. No pós-operatório de queiloplastia, os métodos com sucção são mais benéficos. Porém, no período pós-operatório de palatoplastia há divergências quanto aos métodos mais indicados.

## Introduction

Cleft lip and palate (CLP) are congenital malformations that can affect the lip, the palate, or both,^1^ resulting from errors in the embryonic facial fusion process^2^ due to alterations in the normal development of the primary and/or secondary palate.^3^

With the diagnosis of cleft palate/lip, feeding is a major concern for parents.^4^ Feeding difficulties appear at birth, due to impairment of the suction and swallowing mechanisms resulting from the alteration in the anatomical structures. At this early stage, the priority is monitoring infant nutrition and weight gain.^5^

Surgery is the initial treatment for CLP. Lip repair surgery is recommended by 3 months of life and for the palate, up to 9 or 12 months, as the chronology of procedures admits some variation depending on the specialized center.^6^, ^7^ Adequate nutrition is also important for the child to be able to undergo the cleft repair surgery, i.e., stable weight gain with no health alterations and the capability to safely receive anesthetics.^8^

After the surgical procedure, it is estimated that the child will able to feed with less difficulty, as the oral structures will be repaired. However, in the immediate postoperative period, the conduct regarding feeding also varies, according to the protocols used by the different departments and according to the type of cleft.

Post cleft-lip repair feeding techniques can vary considerably. The recommendations usually range from immediate return to breastfeeding (BF)/bottle to suction abstinence for up to six weeks.^9^ After palatoplasty (palate reconstructive surgery), this divergence is even greater and there are centers that adopt protocols in which bottles and nipples are prohibited for a period of 30 days.^10^

Based on the literature, this systematic review aims to describe studies comparing feeding methods for children with different types of cleft lip/palate in the pre- and postoperative periods, aiming to train parents and professionals for the often difficult task of feeding children with CLP.

## Methods

### Search strategy

The literature search was carried out from January 1, 1990 to August 31, 2015. The search was performed in the PubMed, LILACS, SciELO and Google Scholar databases, as they include most of the publications in this area. This systematic review was conducted according to the PRISMA Statement^11^, ^12^ and registered at PROSPERO (http://www.crd.york.ac.uk/PROSPERO/) under number CRD42014015011.

The search strategy consisted in the following terms (Mesh): (“Cleft Lip” [Mesh] OR “Cleft Palate” [Mesh] OR “Ectodermal Dysplasia” OR “Cleft Lips” OR “Lip, Cleft” OR “Lips, Cleft” OR Harelip OR Harelips OR “Cleft Palates” OR “Palate, Cleft” OR “Palates, Cleft” OR “Cleft Palate, Isolated”) AND (“Feeding Methods” [Mesh] OR “Feeding Method” OR “Method, Feeding” OR “Methods, Feeding” OR “Breast Feeding” [Mesh] OR “Feeding, Breast” OR Breastfeeding OR “Breast Feeding, Exclusive” OR “Exclusive Breast Feeding” OR “Breastfeeding, Exclusive” OR “Exclusive Breastfeeding” OR “Deglutition Disorders” [Mesh] OR “Deglutition Disorder” OR “Disorders, Deglutition” OR “Swallowing Disorders” OR “Swallowing Disorder” OR “Dysphagia” OR “Oropharyngeal Dysphagia” OR “Dysphagia, Oropharyngeal”).

### Selection criteria

Studies that compared feeding methods for children with cleft lip and/or palate and published in English, Portuguese, or Spanish, with level of evidence 1b to 4 according to the criteria proposed by the American Speech-Language-Hearing Association (ASHA)^13^ were included in the review (Table 1). Studies of syndromes associated with the presence of CLP were excluded, as were studies that addressed the use of specific orthopedic methods or those related to surgical techniques. A manual search was performed in the references of the selected articles to identify other possible studies to integrate into the review.

**Table 1:** Levels of scientific evidence according to the criteria proposed by the American Speech-Language-Hearing Association.^13^

Level of evidence	Type of scientific study
1a	Systematic review or meta-analysis of randomized controlled trials
1b	High-quality randomized controlled trials
2a	Systematic review or meta-analysis of non-randomized controlled trials
2b	High-quality non-randomized controlled trials
3a	Systematic review of cohort studies
3b	Cohort studies or low quality randomized controlled trials
4	Studies from clinical outcomes
5a	Systematic review of a case-control study
5b	Case-control studies
6	Series of cases
7	Specialists’ opinion without overt critical assessment

### Data analysis

Two researchers (GAD and RBR) independently reviewed the titles and abstracts of all selected articles to assess whether the studies would be eligible for inclusion in the review. The selected articles were read in full to confirm eligibility and to extract data. Disagreements were resolved by discussion between the two researchers. When necessary, a third reviewer (MCAFC) was consulted. When abstracts did not provide sufficient information, the full text of the article was read for the assessment.

The following information was extracted from each study: year of publication, first author's name, type of study, population, compared feeding methods, number of subjects per group, and assessed parameters.

## Results and discussion

**Figure 1:**
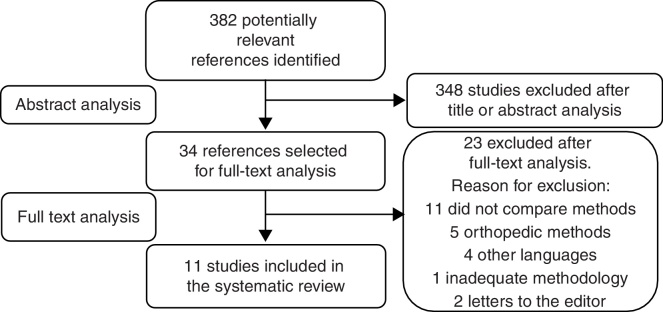
Search strategy.

The search strategy performed to select the studies included in this review is shown in Fig. 1. The initial search identified 382 articles as potentially eligible. After evaluating the title and abstract 348 articles were excluded, as they did not compare feeding methods for children with CLP. Full reading of the 34 remaining studies was performed and of these, 11 studies were selected to integrate the systematic review (seven with level of evidence 1b, three with 3b, and one level 4^13^).

The age range of the studied population was between 0 and 18 months at the start of the studies; however, the follow-up duration was variable. The sample size of the assessed groups ranged from 18 to 96 subjects/observations per group.

The studies addressed 16 different situations of feeding methods or method associations. The main characteristics of the feeding methods of the included studies were: alternative feeding route – nasogastric tube; feeding methods that required suction – bottle and BF; feeding methods without suction – cup, spoon, syringe, and paladai (a shallow cup with a spout popularly used in India).

The parameters evaluated in the studies were: ingested food acceptance and volume; feeding performance; time and complications during feeding; growth and nutritional gain; clinical analysis exams; pain; need for sedation and/or analgesia; dehiscence, presence of fistula and other complications in the surgical wound; hospital length of stay and costs.

Table 2 depicts the characteristics of two studies that compared feeding methods in the preoperative period and one that integrated the pre- and postoperative periods.

**Table 2:** Characterization of studies comparing feeding methods in the preoperative period of surgical repair.

Year Author	LE	*n*/group	Age range	Cleft type	Methods	Assessed parameters	Results
1999 Shaw	1b	52/49	NB	Lip and/or palate	Rigid bottle *vs.* squeezable bottle	Weight; length; head circumference.	Squeezable bottle: beneficial effect on weight gain and head circumference.
2011 Ize-Iyamu	1b	38/19	0–14 w	Involving the palate	Syringe *vs.* cup and spoon	Time of feeding; efficiency (presence of food escape and/or regurgitation); weight gain.	Syringe: higher volume of food and shorter feeding time, less escape and regurgitation and increase in weight gain.
2015 Ravi	3b	50/50/50	2–12 m	Lip and palate	Paladai *vs.* bottle *vs.* spoon	Anthropometrics; weight gain pattern.	Mean weight and mean weight gain velocity: paladai > bottle > spoon; paladai: higher number of well-nourished children until the palate repair surgery; however, after surgery and the start of complementary feeding, the nutritional status of the three groups improved.

*n*, number of subjects or observations; m, months; w, weeks; NB, newborn; LE, American Speech-Language-Hearing Association level of evidence.^13^

Regarding the period prior to the surgical repair of cleft lip and/or palate, two dietary methods were compared: rigid and squeezable bottles. The study evaluated anthropometric data (weight, length, and head circumference) and feeding method reliability (assessed by the need for adjustments to the nipple or change of the feeding method by a health professional). The results showed no differences regarding the anthropometric variables, although an upward trend was observed in weight gain and head circumference in the group fed with squeezable bottle, in addition, the children in this group required fewer nipple modifications and required less professional support and interventions.^14^

The difference between the assisted method (squeezable bottle) and the rigid bottle may be associated with higher volume of food intake and/or lower energy expenditure to extract food from the squeezable bottle, as less effort is required with the assisted method. However, another study evaluated children with cleft palate comparing feeding with rigid and squeezable bottles, with or without the use of shutter, showing no significant differences between the methods regarding caloric intake and growth.^15^

Even though BF is very much encouraged, especially for children with CLP, many of those with cleft palate do not perform well when BF and/or feeding from a bottle before surgical repair. Therefore, Ize-Iyamu et al. compared two alternative methods: syringe *vs.* cup and spoon. The children were followed up weekly aiming to identify the feeding type and difficulties, assess the feeding, evaluate feeding efficiency in relation to food escape and reflow/regurgitation, and assess weight gain between 0 and 14 weeks.

The results showed that the group fed with a syringe had lower feeding time at the 6-week assessment; 100% of the group of infants fed with a cup and spoon had food escape and regurgitation compared with 79% escape and 74% of regurgitation with the syringe at 6 weeks. Moreover, those fed a combination of breast milk and formula using a syringe showed a significant increase in weight gain between the 10th and the 14th week. Thus, the authors reported that feeding with a syringe was a practical, easy-to-perform method, with greater administered volume, less time spent with feeding, and less food escape and regurgitation as well as significant weight gain.^16^

An alternative method, although less well known, was studied by Ravi et al., who compared the impact of feeding with a paladai, a bottle, and a spoon on the weight gain pattern of children aged 2 months to 1 year. Better results were observed in the group fed with a paladai regarding mean weight, mean weight gain velocity, and number of well-nourished children until the palate surgical repair; however, after surgery and the start of complementary feeding, the nutritional status of the three groups improved.^17^

Although the use of the paladai is not common worldwide, studies assessing less widespread methods are important, as these devices may, alone or together with other methods, facilitate food intake and promote lower energy expenditure for some children with cleft lip and/or palate, thereby contributing to greater weight gain.^18^

Table 3 shows the characterization of four studies that compared feeding methods in the postoperative period of surgical repair of cleft lip and/or palate, isolated lip repair, or lip repair associated or not associated with palatoplasty.

**Table 3:** Characterization of studies comparing methods of feeding in the postoperative period of surgical repair of cleft lip and/or palate, isolated lip repair, or lip associated or not associated with palate.

Year Author	LE	*n*/group	Age range	Cleft	Methods	Assessed parameters	Results
1992 Cohen	3b	40/40	4 d to 12 m	Lip and/or palate	Tube and syringe *vs.* Bottle/BF	Surgical wound dehiscence and fistula; weight gain; nutritional status.	Wound dehiscence and presence of fistula was similar in both groups; better weight gain and nutritional status in the bottle/BF group.
1996 Darzi	1b	20/20	3–6 m	Lip	BF *vs.* spoon	Weight; wound dehiscence and appearance; analgesia and intravenous fluids; hospital costs.	BF: greater weight gain. Spoon: greater need for analgesia/sedation and intravenous fluids for a longer period of time and higher hospital costs.
2005 Assunção	1b	23/22	3–13 m	Involving lip	Bottle and spoon *vs.* spoon	Anthropometrics; caloric intake; clinical analysis tests; wound complications.	Similar results with both methods, but with better acceptance of food with bottle and spoon.
2013 Augsornwan	1b	96/96	3–6 m	Involving lip	BF/bottle *vs.* spoon/syringe	Wound complications; parental satisfaction.	Similar results regarding wound dehiscence; parents more satisfied with BF/bottle.

*n*, number of subjects or observations; d, days; m, months; BF, breastfeeding; tube, feeding tube; LE, American Speech-Language-Hearing Association level of evidence.^13^

The use of methods with suction in the postoperative period is a controversial issue. Therefore, Cohen et al. compared the use of feeding tube or syringe *vs.* BF or bottle after lip and/or palate repair surgery. The study evaluated wound dehiscence, presence of oronasal fistula, weight gain, and nutritional status. The results regarding wound dehiscence and the presence of fistula were similar in both groups. However, at the anthropometric and nutritional assessment, better weight gain and nutritional status was observed in the bottle and BF group.^19^

Nonetheless, this study did not perform statistical comparisons of these variables, as they were assessed subjectively by the general impression of observers and nursing staff. Due to methodological and ethical issues, such studies are usually impossible to perform blinded, and observations of the staff were potentially biased.

When comparing BF with spoon feeding regarding weight and surgical wound appearance and dehiscence after lip repair, the results showed that breastfed children had significantly greater weight gain and slightly shorter length of hospital stay. Children fed with a spoon were more irritable, required more analgesic drugs or sedation, and had higher hospital costs. Additionally, there was one case of wound dehiscence and one of scar hypertrophy in spoon-fed group.^20^

A similar study assessing bottle and spoon *vs*. spoon feeding after lip repair showed very similar results between the groups; however, there was greater acceptance of feeding in the group using the bottle and the spoon. Also, infants fed only with a spoon showed irritability and discomfort due to the abrupt change in the type of feeding, which before surgery was performed with the bottle.^21^

When comparing BF/bottle *vs.* spoon/syringe regarding wound dehiscence and parental satisfaction in the postoperative lip repair surgery, this review observed similar results for wound dehiscence; however, parents were more satisfied and relaxed when feeding their children through BF/bottle.^22^

These studies showing favorable results for the feeding method with suction corroborate another study that found that infants submitted to lip repair surgery and fed by bottle showed no adverse effects to the surgery.^9^ The unfavorable results for the methods without suction (spoon and syringe) may be associated with sucking deprivation in infants, which makes them more irritated and, consequently, more agitated and tearful, which affects recovery and food intake, in addition to the fact that lip movement while crying could damage the surgical wound.

Suction is essential to infants, because in addition to being a source of food, it is a comforting factor^23^ and promotes bonding between mother and child, as well as the development of oral motor skills.^24^ Therefore, feeding without restrictions after surgical lip repair is becoming the standard care,^25^ as it has shown better results and lower complication rates.^20^, ^21^, ^22^

However, among the primary surgeries, palate repair is the most invasive and is associated with greater difficulty in accepting oral feeding adequately, which can interfere with the child's weight gain.^19^, ^26^ Additionally, it is traditionally suggested that the bottle should not be used soon after palatoplasty, because inappropriate negative pressure on the suture line may adversely affect the results.^27^

Table 4 summarizes four studies that compare feeding methods in the postoperative period of palate repair surgery, associated or not associated with lip repair.

**Table 4:** Characterization of studies comparing feeding methods in the postoperative period of surgical repair of cleft palate associated or not associated with lip repair.

Year Author	LE	*n*/group	Age range	Cleft palate and/or lip	Methods	Assessed parameters	Results
2009 Kent	3b	34/34	6–12 m	Involving the palate	Feeding tube *vs.* bottle	Feeding; analgesia/pain; hospital length of stay.	Tube: less need for analgesia and length of hospital stay. Bottle: more food rejection, pain, team concerns, difficulty in offering medication and more frequent and prolonged feeding.
2009 Kim	1b	42/40	4–25 m	Involving the palate	Bottle *vs.* spoon, cup and syringe	Complications; use of sedation; oral ingestion; weight gain.	Complications: use of sedation and weight gain similar in both groups. Greater oral intake in the bottle group on the 6th day PO.
2013 Hughes	1b	18/23	5–10 m	Involving the palate	Tube *vs.* OF	Analgesia/pain; intravenous fluids and enteral feeding were administered.	Number of painful episodes and need for morphine administration similar in both groups. Feeding volume was higher in tube group and greater need for intravenous fluid in the OF group.
2013 Trettene	4	88/88	11–18 m	Involving the palate	Cup *vs.* Spoon	Positioning, coughing, choking, escape, feeding time and accepted volume; caregiver safety.	Spoon: less food escape through the lip cleft and greater volume of food received.

*n*, number of subjects or observations; m, months; PO, postoperative; tube, feeding tube; OF, oral feeding; LE, American Speech-Language-Hearing Association level of evidence.^13^

Two studies compare an alternative feeding route or oral/suction method in the first 24 hours of postoperative palate repair surgery. The first study assessed feeding, analgesia, and time of hospital stay of infants fed with a bottle and through a nasogastric tube. The study showed that those fed through the nasogastric tube were more stable, required less analgesia, and were discharged earlier from the hospital. The parents of these infants were more relaxed, knowing that their child was fed and had adequate analgesia, whereas the nurses believed they were able to provide better quality of care. Conversely, the group fed by bottle showed higher feeding rejection, pain, concern from the care team, difficulty receiving medication, and more frequent and prolonged feeding.^28^

The second study evaluated the use of morphine, number of painful episodes, administered intravenous fluid volume, and enteral feeding. The results showed that both the number of painful episodes and the use of morphine were similar in the feeding tube group and the oral group; however, the received food volume was higher in feeding tube group and there was a greater need for intravenous fluid in the oral feeding group.^29^

Both studies by Kent et al. and Hughes et al. assessed pain parameters based on the Face, Legs, Activity, Cry, Consolability (FLACC) scoring system, measured by observations of the nursing staff in the first 24 hours after palate repair surgery.^30^ One of the possible limitations of both studies was the lack of blinding. To correct this, the use of alternative feeding routes would be required for all the children, to maintain the evaluators blinded to the feeding method; however, the feeding tube may damage the palate repair and cause pain and this could be one more confounding factor to the results.^29^

On the other hand, different results were obtained when comparing infants fed with a bottle *vs.* those fed with a spoon, cup and syringe for six days post-palatoplasty. The parameters analyzed were: complications (bleeding, breathing problems, wound dehiscence, and oronasal fistula), frequency of sedation use, oral intake, and weight gain. The rate of overall complications, use of sedation, and weight gain were similar in both groups; however, on the sixth post-operative day, oral intake was greater in the bottle group. According to the authors, these results suggest that the bottle can be introduced during the immediate postoperative period, as the feeding method did not affect the immediate postoperative course of palate repair surgery.^31^

The results of the study by Kim et al. regarding the surgical procedure are different from the studies of Kent et al. and Hughes et al. This might have occurred because the study by Kim et al. had a relatively homogeneous sample of patients regarding the extent of the cleft, used a standard technique for the closing of the palate, and the surgery was performed by a single surgeon. For example, the occurrence of fistulas seems to be related to cleft severity^32^, ^33^ and rarely with the technique employed for the palate closure.^32^ Additionally, the studies by Kent et al. and Hughes et al. evaluated only the first 24 hours, while the study by Kim et al. follows the first six postoperative days. Therefore, it can be assumed that tube feeding would be a more effective method during the first hours and the bottle could be included in the diet on the following days.

Another important factor to be addressed is that different protocols are used in different hospitals, as some institutions prohibit the use of bottles and nipples for a certain period after palate repair surgery. Therefore, the study by Trettene et al. compares feeding with a cup and a spoon in the immediate postoperative period of palatoplasty. This study assessed 44 binomials by caregivers for four consecutive times, intercalating the feeding method. Positioning, coughing, choking, food escape through the lip commissure, feeding time, accepted volume, and safety reported by the caregiver were analyzed.

The results demonstrate that the technique that uses the spoon showed less food escape through the lip commissure, higher food volume acceptance, and children submitted to full palatoplasty had less frequent cough episodes during feeding.^10^ Another positive factor regarding the use of the spoon is the higher degree of oral stimulation and the promotion of muscle contraction and nerve ending stimulation when compared to the cup.^34^, ^35^

Although there are studies comparing similar feeding methods, it was not possible to perform a meta-analysis of the results shown in this systematic review. Studies demonstrated very different methodologies and, above all, very heterogeneous parameters were evaluated, which prevents the equivalence of studies, a fundamental characteristic for the viability of the meta-analysis.

## Conclusion

Feeding through methods with suction is possible and appropriate for children with CLP before the surgical repair, particularly those with isolated pre-foramen clefts, as they are the cases with the greatest chances of success. However, according to the results of the studies, the use of alternative methods such as squeezable bottle, syringe, and paladai may be beneficial in certain cases.

In the postoperative period of lip repair surgery, feeding methods with suction seem to be more beneficial and do not show major complications after surgery. However, regarding the postoperative period of palate repair surgery, there are divergences on the most suitable feeding method, ranging from total interruption of oral feeding for at least 24 hours, suction method deprivation, and feeding method with unrestricted suction. More studies on feeding methods, particularly in the postoperative period of palate repair surgery, are required.

## Conflicts of interest

The authors declare no conflicts of interest.

## References

[1] Jesus M.S.V., Penido F.A., Valente P., Jesus M., Di Ninno C. (2009). Fissura labiopalatina.

[2] Cardim V.L., Altmann E. (2005). Fissuras labiopalatinas.

[3] Marques R.M.F., Lopes L.D., Khoury R.B.F., Altmann E. (2005). Fissuras labiopalatinas.

[4] Araruna RdC, Vendruscolo D.M. (2000). Nutrition of children with cleft lip and cleft palate, a bibliographic study. Rev Lat Am Enfermagem.

[5] Amstalden-Mendes L.G., Magna L.A., Gil-da-Silva-Lopes V.L. (2007). Neonatal care of infants with cleft lip and/or palate: feeding orientation and evolution of weight gain in a nonspecialized Brazilian hospital. Cleft Palate Craniofac J.

[6] Bertier C.E., Trindade I.E.K., Silva Filho O.G., Trindade I.E.K., Silva Filho O.G. (2007). Fissuras labiopalatinas: uma abordagem interdisciplinar.

[7] Martins D.M.F.S., Ferreira L.M. (1995). Manual de cirurgia plástica.

[8] Wyszynski D.F. (2002).

[9] Skinner J., Arvedson J.C., Jones G., Spinner C., Rockwood J. (1997). Post-operative feeding strategies for infants with cleft lip. Int J Pediatr Otorhinolaryngol.

[10] Trettene Ados S., Mondini C.C., Marques I.L. (2013). Feeding children in the immediate perioperative period after palatoplasty: a comparison between techniques using a cup and a spoon. Rev Esc Enferm USP.

[11] Liberati A., Altman D.G., Tetzlaff J., Mulrow C., Gotzsche P.C., Ioannidis J.P. (2009). The PRISMA statement for reporting systematic reviews and meta-analyses of studies that evaluate healthcare interventions: explanation and elaboration. BMJ.

[12] Stroup D.F., Berlin J.A., Morton S.C., Olkin I., Williamson G.D., Rennie D. (2000). Meta-analysis of observational studies in epidemiology: a proposal for reporting. Meta-analysis of Observational Studies in Epidemiology (MOOSE) group. JAMA.

[13] Mullen R. The state of the evidence: ASHA develops levels of evidence for communication sciences and disorders. March 2007. [cited 15 Sept 2015]. Available from: http://www.asha.org.

[14] Shaw W.C., Bannister R.P., Roberts C.T. (1999). Assisted feeding is more reliable for infants with clefts – a randomized trial. Cleft Palate Craniofac J.

[15] Brine E.A., Rickard K.A., Brady M.S., Liechty E.A., Manatunga A., Sadove M. (1994). Effectiveness of two feeding methods in improving energy intake and growth of infants with cleft palate: a randomized study. J Am Diet Assoc.

[16] Ize-Iyamu I.N., Saheeb B.D. (2011). Feeding intervention in cleft lip and palate babies: a practical approach to feeding efficiency and weight gain. Int J Oral Maxillofac Surg.

[17] Ravi B.K., Padmasani L.N., Hemamalini A.J., Murthy J. (2015). Weight gain pattern of infants with orofacial cleft on three types of feeding techniques. Indian J Pediatr.

[18] Di Ninno C.Q.M.S., Moura D., Raciff R., Machado S.V., Rocha C.M.G., Norton R.C. (2011). Aleitamento materno exclusivo em bebês com fissura de lábio e/ou palato. Rev Soc Bras Fonoaudiol.

[19] Cohen M., Marschall M.A., Schafer M.E. (1992). Immediate unrestricted feeding of infants following cleft lip and palate repair. J Craniofac Surg.

[20] Darzi M.A., Chowdri N.A., Bhat A.N. (1996). Breast feeding or spoon feeding after cleft lip repair: a prospective, randomised study. Br J Plast Surg.

[21] Assuncao A.G., Pinto M.A., Peres S.P., Tristao M.T. (2005). Immediate postoperative evaluation of the surgical wound and nutritional evolution after cheiloplasty. Cleft Palate Craniofac J.

[22] Augsornwan D., Surakunprapha P., Pattangtanang P., Pongpagatip S., Jenwitheesuk K., Chowchuen B. (2013). Comparison of wound dehiscence and parent's satisfaction between spoon/syringe feeding and breast/bottle feeding in patients with cleft lip repair. J Med Assoc Thai.

[23] International LLL. Breastfeeding a baby with cleft lip or cleft palate. In: International LLL, editor. Schaumburg, IL, 2004.

[24] Kummer A.W. (2008).

[25] Noordhoff M.S., Chen P.K., Mathes S.J. (2006).

[26] Martin V., Watson A.C.H., Sell D.A., Grunwell P. (2005). Tratamento de fissura labial e fenda palatina.

[27] Randall P., LaRossa D., McCarthy J.G. (1990).

[28] Kent R., Martin V. (2009). Nasogastric feeding for infants who have undergone palatoplasty for a cleft palate. Paediatr Nurs.

[29] Hughes J., Lindup M., Wright S., Naik M., Dhesi R., Howard R. (2013). Does nasogastric feeding reduce distress after cleft palate repair in infants?. Nurs Child Young People.

[30] Merkel S., Voepel-Lewis T., Malviya S. (2002). Pain assessment in infants and young children: the FLACC scale. Am J Nurs.

[31] Kim E.K., Lee T.J., Chae S.W. (2009). Effect of unrestricted bottle-feeding on early postoperative course after cleft palate repair. J Craniofac Surg.

[32] Cohen S.R., Kalinowski J., LaRossa D., Randall P. (1991). Cleft palate fistulas: a multivariate statistical analysis of prevalence, etiology, and surgical management. Plast Reconstr Surg.

[33] Phua Y.S., de Chalain T. (2008). Incidence of oronasal fistulae and velopharyngeal insufficiency after cleft palate repair: an audit of 211 children born between 1990 and 2004. Cleft Palate Craniofac J.

[34] Altmann E.B.C., Vaz A.C.N., Paula M.B.S.F., Khoury R.B.F., Altmann E. (1997). Fissuras lábiopalatinas.

[35] Silva E.B., Rocha C.M.G., Lage R.R., Jesus M.S.V., Di Ninno C.Q.M.S. (2009). Fissura labiopalatina: fundamentos para a prática fonoaudiológica.

